# A System of Medicine

**Published:** 1911-03

**Authors:** 


**A System of Medicine.** By Many Writers. Edited by Sir Clifford Allbutt, K.C.B., M.A., M.D., and Humphry Davy Rolleston, M.A., M.D. Vol. VII. **Diseases of the Muscles, the Trophoneuroses, Diseases of the Nerves, Vertebral Column and Spinal Cord.** Pp. XVI., 900. London: Macmillan & Co. Limited. 1910.

This volume includes parts of the old Volumes VI and VII, and one article, that on "Herpes Zoster," from Volume VIII. Many sections have been more or less rewritten, and several entirely new ones added, such as those on "Amyotonia Congenita" (Dr. J. Collier), "Myasthenia Gravis" (Dr. F. Buzzard), "Family Periodic Paralysis" (Professor J. Michell Clarke), "Syphilis" (Dr. Wilfred Harris), and "Tumours ot the Cord" (Dr. E. Bramwell). A section on "Motor Neuron Disease" replaces the former articles on "Chronic Poliomyelitis" and "Amytrophic Lateral Sclerosis."

The diseases of muscles and the trophoneuroses occupy the two first sections of the volume, otherwise devoted to nerve diseases, and there is iundoubtedly a convenience in this arrangement, though the present tendency in several of these disorders is to refer them entirely away from nerve changes. Thus Sir Thomas Barlow has come to look on erythromelalgia as due to vascular disease, and the nerve changes and vaso-motor states as secondary, though he reverses the process in Raynaud's disease.

Buzzard points out that the interest in myasthenia gravis in this country only dates from Campbell and Bramwell's monograph in 1900, and thinks it is due to glandular changes. He shows how often it is associated with Graves's disease, and that the most common finding is deposits of mononuclear cells in the muscles and elsewhere. Another most interesting disease is family periodic paralysis. Professor Clarke's picture of it seems to us to present striking analogies to migraine, though sensation and consciousness are unaffected. Temporary attacks of paralysis of the muscles, except those supplied by the cranial nerves, occur from time to time, especially in early adult life, and last one or two days. They may be brought on by fatigue, excitement or overfeeding, and are sometimes preceded by prodromal sensations.

Nearly one hundred and twenty pages are devoted to a most instructive survey of the nervous system and its pathology, by Dr. F. W. Mott, which will be found an invaluable exposition of recent researches on the subject. The story of the discovery of the neurons and their processes leads up to the discussion of how they are affected by disease. They cannot be regenerated if destroyed, and are both excitable cells and conductors of energy. The nerves which are processes of the cells are subject to similar oxidation processes. Dr. Mott goes on to show how changes in the neurons, like those produced in disease, have been produced and studied by either cutting off the blood supply or by surrounding them with an atmosphere of N or O, and then compares with these the actual results found in various pathological states.

In the discussion of the linking together of the nervous system we get an interesting account of reflex action and of pain as a psychical adjunct to a protective reflex, introducing us to the work of Sherrington, Pavlov, and Richet. The paper concludes with an account of the cerebro-spinal fluid as the medium of gaseous exchange, and of the possible source of energy to be found in the dextrose it contains. He insists that a key to many pathological changes in the brain can be found in the presence of substances such as choline and cholesterol in this fluid.

Starr has added to his account of acute anterior poliomyelitis a notice of recent epidemics and some of Flexner's discoveries as to the organism involved. In caisson disease Leonard Hill describes recent studies on the physiological conditions of work in caissons and in diving under pressures up to 68 lb. on the square inch, with the consequent increased supply of oxygen. His experiments show that the symptoms are due to the tendency of the nitrogen absorbed to escape irregularly from the various tissues as bubbles in the blood when the pressure is suddenly removed. Gradual decompression prevents this. Admiralty divers are decompressed in several stages. The author finds that one long stay at 35 lb. to the inch is sufficient, but the shifts of work should not be too long, and old or fat men should not be employed. The mortality in recent operations has been greatly reduced, and immediate recompression is advised if a man is attacked.

